# Bispecific human IL2‐CCR4 immunotoxin targets human cutaneous T‐cell lymphoma

**DOI:** 10.1002/1878-0261.12653

**Published:** 2020-03-13

**Authors:** Haoyu Wang, Zhaohui Wang, Huiping Zhang, Zeng Qi, Ariel C. Johnson, David Mathes, Elizabeth A. Pomfret, Erin Rubin, Christene A. Huang, Zhirui Wang

**Affiliations:** ^1^ Division of Plastic and Reconstructive Surgery Department of Surgery School of Medicine University of Colorado Denver Aurora CO USA; ^2^ Division of Transplant Surgery Department of Surgery School of Medicine University of Colorado Denver Aurora CO USA; ^3^ Center for Transplantation Sciences Massachusetts General Hospital and Harvard Medical School Boston MA USA; ^4^ College of Animal Science and Veterinary Medicine Shanxi Agricultural University Taigu China; ^5^ Transplantation Pathology Department of Pathology School of Medicine University of Colorado Denver Aurora CO USA

**Keywords:** CCR4, cutaneous T‐cell lymphoma, IL2, immunotoxin, Ontak®

## Abstract

The majority of clinically diagnosed cutaneous T‐cell lymphomas (CTCL) highly express the cell‐surface markers CC chemokine receptor 4 (CCR4) and/or CD25. Recently, we have developed diphtheria toxin‐based recombinant Ontak®‐like human IL2 fusion toxin (IL2 fusion toxin) and anti‐human CCR4 immunotoxin (CCR4 IT). In this study, we first compared the efficacy of the CCR4 IT vs IL2 fusion toxin for targeting human CD25^+^CCR4^+^ CTCL. We demonstrated that CCR4 IT was more effective than IL2 fusion toxin. We further constructed an IL2‐CCR4 bispecific IT. The bispecific IT was significantly more effective than either IL2 fusion toxin or CCR4 IT alone. The bispecific IT is a promising novel targeted therapeutic drug candidate for the treatment of refractory and recurrent human CD25^+^ and/or CCR4^+^ CTCL.

## Graphical abstract

We have developed an IL2‐CC chemokine receptor 4 (CCR4) bispecific immunotoxin (IT). The bispecific IT was significantly more effective than either IL2 fusion toxin or CCR4 IT alone. The bispecific IT is a promising novel targeted therapeutic drug candidate for the treatment of refractory and recurrent human CD25^+^ and/or CCR4^+^ cutaneous T‐cell lymphoma.

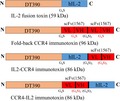

**Abbreviations**
BiscFv: bivalent single‐chain variable fragment
CCR4: CC chemokine receptor 4
CTCL: cutaneous T‐cell lymphoma
DT: diphtheria toxin
FDA: Food and Drug Administration
G_4_S: four glycine residues and one serine residue
His: histidine
IC_50_: half‐maximal inhibitory concentration
IP: intraperitoneal injection
IT: immunotoxin
IV: intravenous injection
*K*_d_: dissociation constant
mAb: monoclonal antibody
MFI: mean fluorescence intensity
mono: monovalent
*NSG*: NOD/SCID IL2 receptor γ^−^^/^^−^
scFv: single‐chain variable fragment
SD: standard deviation
VH: heavy chain variable domain
VL: light chain variable domain

## 1 Introduction

Cutaneous T‐cell lymphoma (CTCL) is a type of extranodal non‐Hodgkin's lymphoma characterized by skin lesions resulting from infiltration of malignant T lymphocytes. The two main forms of CTCL are mycosis fungoides and Sezary syndrome (Duvic *et al.*, 2013; Prince *et al.*, 2010). Treatment of early‐stage CTCL (IA–IIA) primarily involves the use of skin‐directed therapies including topical corticosteroids, phototherapy (psoralen with UVA or UVB), topical chemotherapy, topical bexarotene, and radiotherapy including localized radiation and total skin electron beam therapy (Devata and Wilcox, 2016; Tarabadkar and Shinohara, 2019; Zinzani *et al.*, 2016). Refractory early‐stage and advanced‐stage CTCL (IIB–IV) requires systemic treatment using such as bexarotene, vorinostat, Ontak® (denileukin diftitox; Eisai Medical Research, Inc., Ridgefield Park, NJ, USA), romidepsin, brentuximab, and recently approved mogamulizumab (Alpdogan *et al.*, 2019; Devata and Wilcox, 2016; Zinzani *et al.*, 2016). However, their objective response rate remains approximately 30% (Alpdogan *et al.*, 2019; Ohmachi *et al.*, 2018). There is still an unmet medical need for development of novel targeted therapeutic drugs for relapsed or recurrent CTCL patients.

CC chemokine receptor 4 (CCR4) is overexpressed in CTCL skin lesions at all stages of disease and is recognized as a promising novel therapeutic target for CTCL (Ferenczi, *et al.*, 2002; Sugaya *et al*, 2015). Immunohistochemical CCR4 expression is 14–97% in the skin of CTCL patients and 90–100% in the clinical trials enrolling patients with relapsed diseases. CCR4 is detectable in almost all of the CTCL cases involving the blood using flow cytometry, and percentages of CCR4‐positive cells range from 31% to 97%. This is significantly higher than among healthy individuals (27%) (Ollila *et al.*, 2019). Recently, we have developed a recombinant anti‐human CCR4 immunotoxin (IT) for targeting CCR4^+^ tumors and Tregs using a unique diphtheria toxin (DT)‐resistant yeast *Pichia pastoris* expression system (Wang *et al.*, 2015). The efficacy for targeting CCR4^+^ tumors was characterized using a CCR4^+^ T‐cell acute lymphoblastic leukemia tumor‐bearing immunodeficient NOD/SCID IL2 receptor γ^−/−^ (*NSG*) mouse model (Wang *et al.*, 2015). The CCR4^+^ Treg depletion efficacy was demonstrated using naïve cynomolgus monkeys (Wang *et al.*, 2018; Wang *et al.*, 2016).

Although CD25 is expressed in fewer CTCL cases than CCR4, it is recognized as an important therapeutic target. Nichols *et al. *(1997) reported that CD25 is expressed in ~ 50% of CTCL cases. CD25 expression is found more commonly in lesions from advanced‐CTCL patients (Talpur *et al.*, 2006). Ontak® (denileukin diftitox; Eisai Medical Research, Inc.) is a truncated DT‐based recombinant human IL2 fusion toxin expressed in *Escherichia coli*. It was approved by the Food and Drug Administration (FDA) for treatment of patients with persistent and recurrent CD25^+^ CTCL. The overall response rates of Ontak® range from 30% to 50% (Duvic *et al.*, 2013; Prince *et al.*, 2010). However, Ontak® was discontinued clinically due to production issues related to *E. coli* expression and purification. E7777 is a new version of Ontak® with improved purity and a high percentage of active monomer. It is currently under phase III clinical trial (Ohmachi *et al.*, 2018). We have developed an Ontak®‐like human IL2 fusion toxin (IL2 fusion toxin) using the same DT‐resistant yeast *P. pastoris* expression system (Liu *et al.*, 2003) and characterized its efficacy *in vitro* and *in vivo* (Peraino *et al.*, 2014; Wang *et al.*, 2017).

In this study, we compared the efficacy of the CCR4 IT vs IL2 fusion toxin to human CD25^+^CCR4^+^ CTCL. The result demonstrated that the CCR4 IT was more effective than the IL2 fusion toxin. We further constructed an IL2‐CCR4 bispecific IT. The bispecific IT was even more effective than either IL2 fusion toxin or CCR4 IT alone.

## 2 Materials and methods

### 2.1 Antibodies and cell line

Antibodies used in this study are listed in Table 1. Human CD25^+^CCR4^+^ T‐cell lymphoma cell line Hut102/6TG (Williams *et al.*, 1990) was generously provided by Robert Harrison of Anjin Group, Inc., Boston, MA, USA. Human T‐lymphocyte cell line Jurkat, clone E6‐1 (ATCC TIB‐152); human CCR4^+^ acute lymphoblastic leukemia cell line CCRF‐CEM (ATCC CCL‐119, Manassas, VA, USA); and human CD25^+^ lymphoma cell line SR (ATCC CRL‐2262) were purchased from ATCC.

**Table 1:** Antibodies used in this study.

Antibody name	Clone	Source	Cat#
Fluorescein‐mouse anti‐human/rat CCR4	205410	R&D Systems (Minneapolis, MN, USA)	FAB1567F
Mouse IgG2B fluorescein isotype control	133303	R&D Systems	IC0041F
FITC‐mouse anti‐human CD25	BC96	BioLegend (San Diego, CA, USA)	302604
FITC‐mouse IgG1, κ isotype control	MOPC‐21	BioLegend	400108
Phycoerythrin‐streptavidin		BioLegend	405204
Propidium iodide		Sigma (St. Louis, MO, USA)	81845
7‐Aminoactinomycin D		Sigma	A9400

### 2.2 Immunotoxin expression and purification

#### 2.2.1 IL2‐CCR4 bispecific immunotoxin construction

**Fig. 1:**
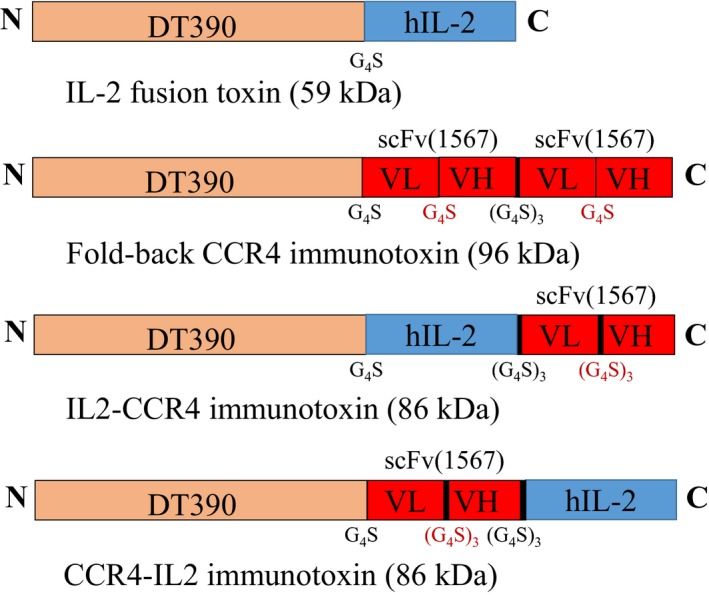
Schematic diagrams of (a) Ontak®‐like human IL2 fusion toxin; (b) single‐chain foldback diabody anti‐human CCR4 IT; (c) IL2‐CCR4 bispecific IT; and (d) CCR4‐IL2 bispecific IT.

Bivalent anti‐human CCR4 IT [DT390‐BiscFv(1567)‐6xHis] in pwPICZalpha (Wang *et al.*, 2015) was digested using *Nco*I and *Bam*HI and separated with DNA agarose gel. The large band (~ 4.35 kb) was cut out and extracted as vector. Bivalent human IL2 fusion toxin (DT390‐Bi‐hIL2‐6xHis) developed previously (Peraino *et al*, 2014) was digested using *Nco*I and *Bam*HI. The digestion mixture was separated with DNA agarose gel. The human IL2 (~ 459 bp) carrying *Nco*I and *Bam*HI was cut out and extracted as insert. The prepared human IL2 insert carrying *Nco*I and *Bam*HI was cloned into the prepared pwPICZalpha‐DT390‐scFv(1567) vector (*Nco*I–*Bam*HI digested), yielding the IL2‐CCR4 bispecific IT DNA construct (Fig. 1).

#### 2.2.2 CCR4‐IL2 bispecific immunotoxin construction

Bivalent anti‐human CCR4 IT [DT390‐BiscFv(1567)‐6xHis] in pwPICZalpha (Wang *et al.*, 2015) was digested using *Bam*HI and *Eco*RI and separated with DNA agarose gel. The large band (~ 4.35 kb) was cut out and extracted as vector. Bivalent human IL2 fusion toxin in pwPICZalpha (DT390‐Bi‐hIL2‐6xHis) (Peraino *et al.*, 2014) was digested using *Bam*HI and *Eco*RI. The digestion mixture was separated with DNA agarose gel. The human IL2 (~ 459 bp) carrying *Bam*HI and *Eco*RI was cut out and extracted as insert. The prepared human IL2 insert carrying *Bam*HI and *Eco*RI was cloned into the prepared pwPICZalpha‐DT390‐scFv(1567) vector (*Bam*HI–*Eco*RI digested), yielding the CCR4‐IL2 bispecific IT DNA construct (Fig. 1). The linearized IT DNA construct (IL2‐CCR4 bispecific IT or CCR4‐IL2 bispecific IT) was transformed into DT‐resistant yeast *P. pastoris* strain (Liu *et al.*, 2003) for expression and purification as previously described (Wang *et al.*, 2015). IL2 fusion toxin (Peraino *et al.*, 2014), CCR4 IT (Wang *et al.*, 2015), and C21 IT (truncated diphtheria toxin DT390‐based unrelated IT) were also expressed and purified in our laboratory using the same DT‐resistant yeast *P. pastoris* expression system. Western blot analysis, flow cytometry binding affinity analysis, and *K*_d_ determination were all performed as previously described (Peraino *et al.*, 2014; Wang *et al.*, 2015).

### 2.3 *In vitro* efficacy analysis

*In vitro* efficacy of the ITs against the tumor cell line was assessed using CellTiter‐Glo® Luminescent Cell Viability Assay (Promega, cat# G7571, Madison, WI, USA) as described previously (Zheng *et al.*, 2017). The cell viability assay measures the luminescence produced as a result of ATP production from metabolically active cells. The increasing concentrations of cytotoxic ITs lead to cell death and a corresponding reduction in ATP‐related fluorescence. The luminescence signals were recorded using Wallac Victor2 1420 multilabel counter (Perkin Elmer, Waltham, MA, USA).

### 2.4 *In vivo* efficacy analysis

Human CD25^+^CCR4^+^ CTCL Hut102/6TG tumor‐bearing immunodeficient *NSG* mouse model was employed to assess the *in vivo* efficacy of the ITs as described (Wang *et al.*, 2015). Breeding pairs of immunodeficient *NSG* mice were purchased from Jackson Laboratories (Bar Harbor, ME, USA) and bred in our rodent barrier facilities. All animal care procedures and experiments were approved by the Institutional Animal Care and Use Committee of Massachusetts General Hospital and University of Colorado Anschutz Medical Campus. The *NSG* mice were divided into the following groups: (a) C21 IT group as a negative control (a nonrelated DT‐based IT; *n* = 13); (b) Ontak®‐like human IL2 fusion toxin group (*n* = 12); (c) single‐chain foldback diabody anti‐human CCR4 IT group (*n* = 12); (d) IL2‐CCR4 bispecific IT group (*n* = 12); and (e) CCR4‐IL2 bispecific IT group (*n* = 14). All animals were intravenous (IV) injected at day 0 with 10 million human CD25^+^CCR4^+^ Hut102/6TG tumor cells *via* the tail vein. The IT was intraperitoneal injection (IP) injected from day 4 on at 8.3 × 10^−10^ moles·kg^−1^, once daily for 10 consecutive days. The injected animals were observed daily for signs and symptoms of illness and scored at least twice weekly based on the parameters as previously reported by our laboratory (Peraino *et al.*, 2013; Wang *et al.*, 2015). The animals were humanely euthanized when the score exceeded the limit or the animal lost more than 15% of its pre‐injection body weight.

### 2.5 Pathology analysis

Liver necropsy specimens were obtained surgically on day 21 after animal euthanasia. Tissues were fixed in 10% formalin and embedded in paraffin and subsequently sectioned. Tissues were stained with hematoxylin and eosin for routine light microscopy. Slides were digitalized by Aperio Scanscope (Leica, Allendale, NJ, USA), and images were analyzed at 2× and 30× with aperio imagescope software (Leica).

### 2.6 Statistical analysis

All *P* values were calculated using two‐way ANOVA or log‐rank (Mantel–Cox) test of prism 8 (GraphPad Software Inc., San Diego, CA, USA). *P* < 0.05 was considered as significant. Half‐maximal inhibitory concentration (IC_50_) was determined using nonlinear regression (curve fit) of prism 8 (GraphPad Software Inc.).

## 3 Results

### 3.1 CCR4 immunotoxin is more effective than IL2 fusion toxin to human CD25^+^CCR4^+^ CTCL Hut102/6TG

Previously, we have demonstrated that IL2 fusion toxin was effective in targeting human CD25^+^CCR4^+^ CTCL Hut102/6TG *in vitro* and *in vivo* (Peraino *et al.*, 2014; Wang *et al.*, 2017). In this study, we first analyzed the binding affinity of the CCR4 IT to human CD25^+^CCR4^+^ CTCL Hut102/6TG using flow cytometry. The results showed that biotinylated CCR4 IT bound to human CD25^+^CCR4^+^ CTCL Hut102/6TG in a dose‐dependent manner (Fig. 3A) with a *K*_d_ value of 21.93 nm (Fig. 3B), which was stronger than that of IL2 fusion toxin with *K*_d_ value of 60.38 nm (Fig. 3B). We then performed *in vitro* efficacy comparison of the CCR4 IT vs IL2 fusion toxin to human CD25^+^CCR4^+^ CTCL Hut102/6TG using luminescent cell viability assay. As shown in Fig. 4 and Table 2, the IL2 fusion toxin (IC_50_ = 1 × 10^−10.5^ m) was more effective than the CCR4 IT (IC_50_ = 1 × 10^−9.8^ m) to human CD25^+^CCR4^+^ CTCL Hut102/6TG. *In vivo* efficacy comparison analysis demonstrated that CCR4 IT prolonged human CD25^+^CCR4^+^ Hut102/6TG‐bearing immunodeficient *NSG* mouse survival significantly more than IL2 fusion toxin with median survival days of 40 vs 33.5 (Fig. 5, Tables 2 and 3).

**Table 2:** *In vitro* and *in vivo* efficacy summary of the tested ITs in this study.

	C21 IT	IL2 IT	CCR4 IT	IL2‐CCR4 IT	CCR4‐IL2 IT
IC_50_ (m)		10^−10.5^	10^−9.8^	10^−11.2^	10^−11.5^
Median survival days	24	33.5	40	57	69

**Table 3:** Survival curve log‐rank (Mantel–Cox) test.

	C21 IT	IL2 IT	CCR4 IT	IL2‐CCR4 IT	CCR4‐IL2 IT
C21 IT	–	***	***	***	***
IL2 IT	***	‐	***	***	***
CCR4 IT	***	***	–	***	***
IL2‐CCR4 IT	***	***	***	–	**
CCR4‐IL2 IT	***	***	***	**	–

** *P ≤ *0.01

*** *P* ≤ 0.001.

### 3.2 Expression and purification of the IL2‐CCR4 and CCR4‐IL2 bispecific immunotoxins

**Fig. 2:**
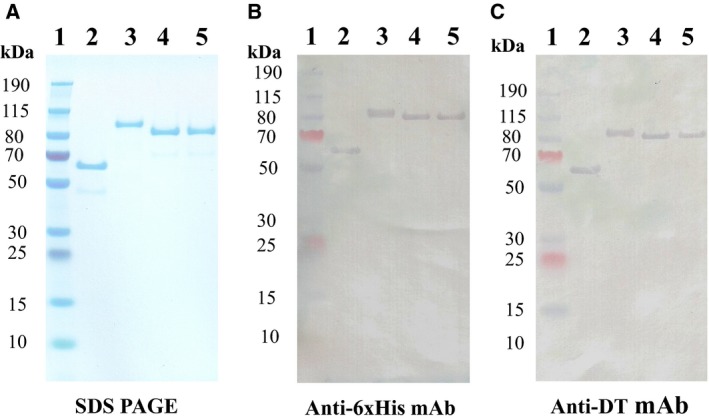
SDS/PAGE and western blot analysis of the bispecific ITs. (A) SDS/PAGE analysis (4–12% NuPAGE; Invitrogen, Carlsbad, CA, USA); the lower molecular weight weak band (~ 45 kDa) in lane 2 is the broken‐down product of the IL2 fusion toxin; (B) western blot analysis using a mouse anti‐His mAb (clone #4A12E4; Invitrogen); (C) western blot analysis using a mouse anti‐DT mAb (clone #3B6; Meridian). Lane 1: protein marker; lane 2: Ontak®‐like monovalent human IL2 fusion toxin (59 kDa); lane 3: single‐chain foldback diabody anti‐human CCR4 IT (96 kDa); lane 4: IL2‐CCR4 bispecific IT (86 kDa); and lane 5: CCR4‐IL2 bispecific IT (86 kDa).

We hypothesized that the IL2‐CCR4 bispecific IT would be more effective than the IL2 fusion toxin or CCR4 IT alone for targeting CD25^+^CCR4^+^ CTCL. To develop the best bispecific IT, we constructed two versions of the bispecific ITs: (a) IL2‐CCR4 bispecific IT; and (b) CCR4‐IL2 bispecific IT (Fig. 1). The bivalent anti‐human CCR4 IT (Wang *et al.*, 2015) was used as a template to construct the IL2‐CCR4 or CCR4‐IL2 bispecific IT by replacing the first or second anti‐human CCR4 scFv using human IL2 (Fig. 1). The bispecific ITs were expressed and purified using the DT‐resistant yeast *P. pastoris* expression system (Liu *et al.*, 2003) as previously described by our laboratory (Wang *et al.*, 2015) (Fig. 2). The final purification yield is ~ 7 mg per liter of the original harvested supernatant. SDS/PAGE and western blot analysis demonstrated that the two versions of the bispecific IT were successfully expressed and purified with expected molecular weight of ~ 86 kDa (Fig. 2A–C).

### 3.3 Bispecific immunotoxin shows higher *in vitro* binding affinity and efficacy compared to the monospecific immunotoxins

**Fig. 3:**
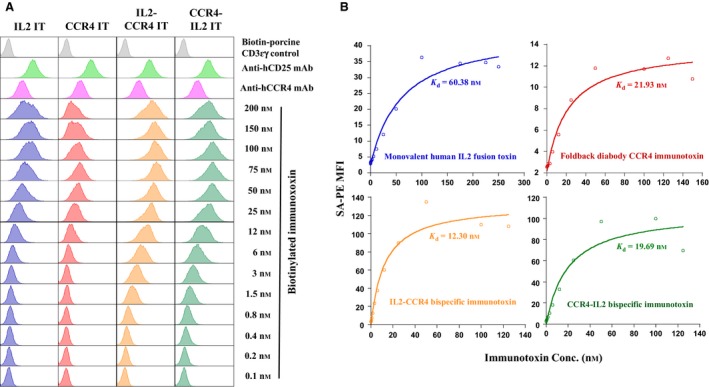
(A) Flow cytometry binding affinity analysis of the biotinylated (a) IL2 fusion toxin alone; (b) CCR4 IT alone; (c) IL2‐CCR4 bispecific IT; and (d) CCR4‐IL2 bispecific IT to human CD25^+^CCR4^+^ Hut102/6TG cells. Fluorescein‐mouse anti‐human/rat CCR4 mAb and FITC‐mouse anti‐human CD25 mAb were used as positive controls. Biotin‐labeled porcine CD3‐εγ (Peraino *et al.*, 2012) was included as a negative control for background due to protein biotinylation. The data are representative of three individual experiments. (B) *K*_d_ determination using flow cytometry and nonlinear least‐squares fit. Mean fluorescence intensity (MFI) was plotted over a wide range of concentrations of biotinylated (a) IL2 fusion toxin alone; (b) CCR4 IT alone; (c) IL2‐CCR4 bispecific IT; and (d) CCR4‐IL2 bispecific IT. The accompanying least‐squares fits are shown based on the hyperbolic equation: *y* = *m*1 + *m*2*m0/(*m*3 + *m*0), where *y* = MFI at the given biotinylated IT concentration, *m*0 = biotinylated IT concentration, *m*1 = MFI of zero biotinylated IT control, *m*2 = MFI at saturation, and *m*3 = *K*_d_.

**Fig. 4:**
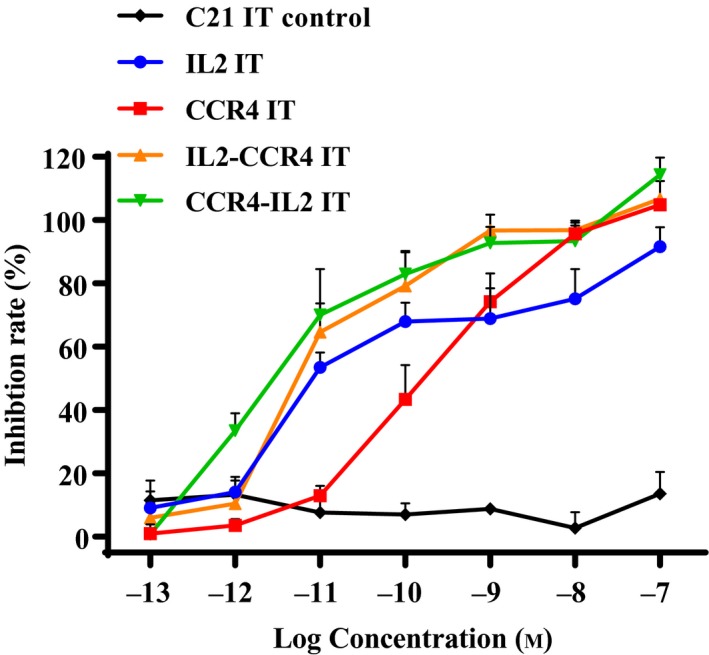
*In vitro* efficacy analysis of the bispecific ITs using CellTiter‐Glo® Luminescent Cell Viability Assay (Promega, cat# G7571) to human CD25^+^CCR4^+^ T‐cell lymphoma cell line Hut102/6TG. (a) C21 IT as negative control (black line); (b) IL2 fusion toxin alone (blue line); (c) CCR4 IT alone (red line); (d) IL2‐CCR4 bispecific IT (orange line); and (e) CCR4‐IL2 bispecific IT (green line). *Y*‐axis: inhibition rate of the cell viability by determining the number of viable cells based on the quantification of the ATP present. *X*‐axis: plated IT concentration. Cycloheximide (1.25 mg·mL^−1^) was used as a positive control. The negative control contained cells without IT. Data were from three independent assays. Statistical analysis was performed using two‐way ANOVA (*n* = 3). Error bars indicate ± SD.

The binding affinity of the biotinylated IL2‐CCR4 or CCR4‐IL2 bispecific IT to human CD25^+^CCR4^+^ Hut102/6TG was analyzed using flow cytometry and found to have a *K*_d_ value of 12.3 nm for IL2‐CCR4 IT and 19.69 nm for CCR4‐IL2 IT (Fig. 3A,B). The bispecific ITs bound stronger than IL2 fusion toxin and CCR4 IT individually (Fig. 3A,B). The *in vitro* efficacy was assessed using luminescence‐based cell viability assay. The bispecific ITs (IC_50_ = 10^−11.2^ m for IL2‐CCR4 IT and IC_50_ = 10^−11.5^ m for CCR4‐IL2 IT) were more potent than either CCR4 IT (IC_50_ = 10^−9.8^ m) or IL2 fusion toxin alone (IC_50_ = 10^−10.5^ m; Fig. 4 and Table 2).

To further characterize the bispecific ITs, we performed *in vitro* flow cytometry binding affinity and luminescence‐based cell viability analysis of the bispecific ITs to (a) human CD25‐ and CCR4‐double‐negative Jurkat cell line; (b) human CD25‐single‐positive SR cell line; and (c) human CCR4‐single‐positive CCL‐119 cell line. As shown in Figs S1A and S2A, no *in vitro* binding or efficacy was observed for the bispecific ITs to CD25‐ and CCR4‐double‐negative Jurkat cells. As shown in Figs S1B and S2B, higher *in vitro* binding and efficacy were observed for the bispecific ITs to CD25‐single‐positive SR cells than those of the IL2 fusion toxin alone. As shown in Figs S1C and S2C, weak *in vitro* binding and efficacy were observed for the bispecific ITs to CCR4 single‐positive CCL‐119 cells. The binding affinity and efficacy were significantly weaker than those of the foldback diabody anti‐human CCR4 IT (Figs S1C and S2C; Wang *et al.*, 2015) and comparable to those of the monovalent anti‐human CCR4 IT (Figs S1D and S2D; Wang *et al.*, 2015). This is because the bispecific ITs only contain one anti‐human CCR4 scFv. Taken together, the bispecific ITs were more effective than IL2 fusion toxin or CCR4 IT alone to CCR4‐ and CD25‐double‐positive or CD25‐single‐positive cells, but less effective than foldback diabody anti‐human CCR4 IT alone to CCR4‐single‐positive cells. Foldback diabody anti‐human CCR4 IT (Wang *et al.*, 2015) is still more effective than the bispecific ITs for targeting CCR4‐single‐positive cells.

### 3.4 Bispecific immunotoxins shows greater tumor response *in vivo* compared to the monospecific immunotoxins

**Fig. 5:**
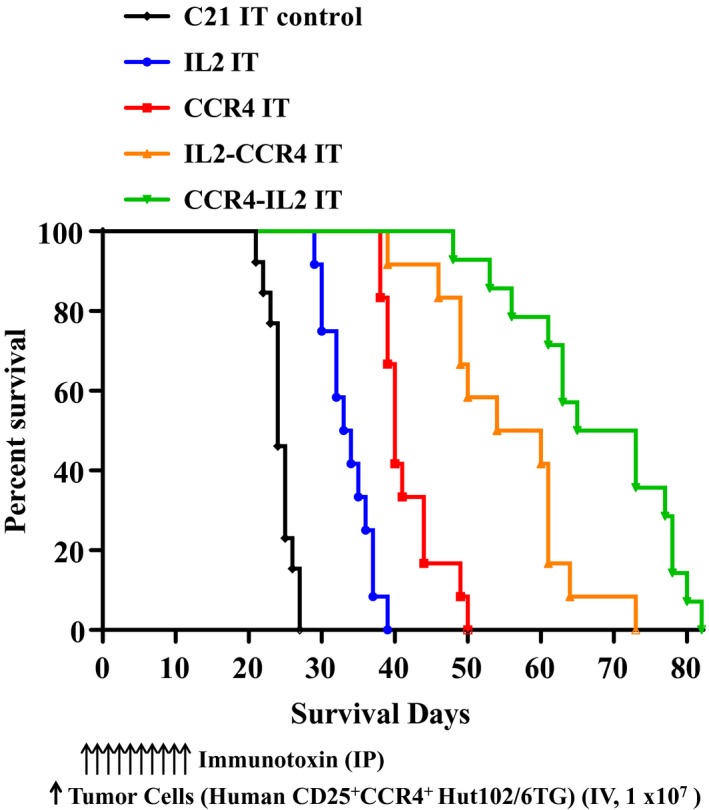
*In vivo* efficacy assessment of the bispecific ITs. *NSG* mice were IV injected with 1.0 × 10^7^ human CD25^+^CCR4^+^ Hut102/6TG cells on day 0 and treated from day 4 on with the IT (IP injection) at 8.3 × 10^−10^ moles·kg^−1^ daily for 10 consecutive days. (a) C21 IT control group (a nonrelated DT390‐based IT as negative control; *n* = 13, black curve) with a median survival time of 24 days; (b) IL2 fusion toxin‐alone group (*n* = 12, blue curve) with a median survival time of 33.5 days; (c) CCR4 IT‐alone group (*n* = 12, red curve) with median survival time of 40 days; (d) IL2‐CCR4 bispecific IT group (*n* = 12, orange curve) with a median survival time of 57 days; and (e) CCR4‐IL2 bispecific IT group (*n* = 14, green curve) with a median survival time of 69 days. The schedule of the IT and tumor cell injection is pictured in the schematic below the survival curve. The vertical arrows indicate the days on which the tumor cells or the ITs were injected. The data are pooled from two separate experiments.

**Fig. 6:**
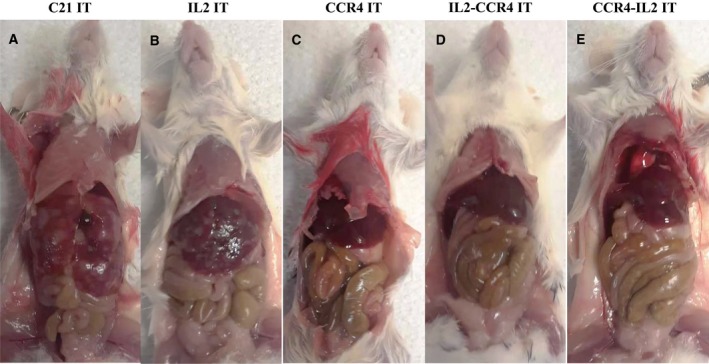
Liver necropsy examination of the representative tumor‐bearing mice at day 21 from (A) the C21 IT group; (B) the IL2 fusion toxin‐alone group; (C) the CCR4 IT‐alone group; (D) the IL2‐CCR4 bispecific IT group; and (E) the CCR4‐IL2 bispecific IT group.

**Fig. 7:**
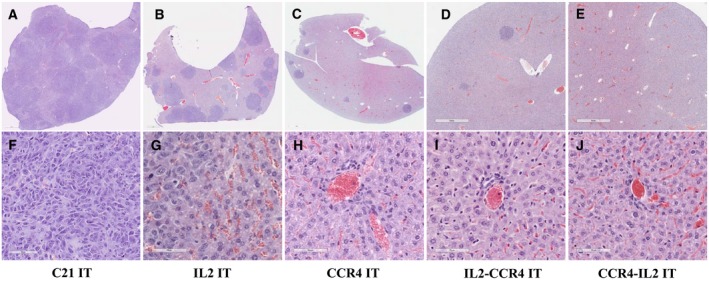
Pathology analysis: (A, F) Liver from a mouse injected with both Hut102/6TG tumor cells and C21 IT (negative control) shows extensive tumor infiltration with replacement of liver parenchyma by tumor cells. (B, G) Liver from a mouse injected with both Hut102/6TG tumor cells and IL2 fusion toxin shows fewer tumor cell areas in the examined section. (C, H) Liver from a mouse injected with both Hut102/6TG and CCR4 IT shows only a few sporadic tumor nodules. (D, E, I, J) Liver from a mouse injected with both Hut102/6TG and IL2‐CCR4 or CCR4‐IL2 bispecific IT shows normal or near‐normal hepatic parenchyma in the examined section. Two tumor cell areas were observed in the examined section of IL2‐CCR4 IT‐treated animal liver (D) and none in CCR4‐IL2 IT‐treated animal liver (E). Scale bars: 1 mm (A–E) and 70 µm (F–J).

*In vivo* efficacy of the IL2‐CCR4 or CCR4‐IL2 bispecific IT was assessed using CD25^+^CCR4^+^ CTCL Hut102/6TG‐bearing immunodeficient *NSG* mouse model. The tumor cells were IV injected on day 0. The ITs were IP injected starting on day 4 for 10 consecutive days. As shown in Fig. 5, the bispecific ITs significantly prolonged median survival from 24 days in the C21 IT control group to 57 and 69 days in the IL2‐CCR4 and CCR4‐IL2 bispecific IT groups, respectively. This median survival was significantly longer than the median survival of 33.5 and 40 days for IL2 fusion toxin and CCR4 IT, respectively (Fig. 5, Tables 2 and 3). As shown in Fig. 6 and Fig. S3, gross examination during necropsy on day 21 revealed enlarged livers with extensive CTCL tumor nodules on the liver surface of C21 IT control animals. The IL2 fusion toxin group had less hepatomegaly with fewer tumor nodules when compared with the C21 IT control group. The CCR4 IT group and the two bispecific IT groups demonstrated normal liver size with few sporadic tumor nodules (Fig. 6 and Fig. S3). Histological evaluation of the liver was performed at day 21 for five representative tumor‐bearing *NSG* mice. For the C21 IT group, hematoxylin and eosin staining revealed replacement of liver parenchyma with extensive tumor nodules (Fig. 7A,F). In the IL2 fusion toxin group, fewer tumor cell areas were seen in the examined section of the liver compared to those of the C21 IT group (Fig. 7B,G). This pathology analysis confirms that the IL2 fusion toxin has efficacy in depletion of human CD25^+^CCR4^+^ tumor cells. Pathology of the CCR4 IT‐alone group showed sporadic tumor cell areas (Fig. 7C,H), demonstrating that CCR4 IT treatment is more effective against the tumor cells than IL2 fusion toxin treatment. In the IL2‐CCR4 IT group (Fig. 7D,I), two tumor cell areas were identified in the examined section of the liver (see Fig. 7D). In the CCR4‐IL2 IT group (Fig. 7E,J), no tumor cell areas were identified in the examined section of the liver (see Fig. 7E), demonstrating that both the IL2‐CCR4 and CCR4‐IL2 bispecific ITs more effectively depleted the human CD25^+^CCR4^+^ tumor cells than IL2 fusion toxin or CCR4 IT. Taken together, these *in vivo* efficacy data consistently demonstrated that CCR4 IT is more effective than IL2 fusion toxin and that the bispecific IT showed more efficacy than either CCR4 IT or IL2 fusion toxin alone.

## 4 Discussion

Mogamulizumab (KW0761), a defucosylated monoclonal antibody (mAb) specific for human CCR4, has been approved for treatment of CCR4^+^ CTCL patients in Japan and the United States (Kim *et al.*, 2018). However, monoclonal antibodies often have poor therapeutic function in tissues such as bone marrow and skin, likely due to lack of accessory cells from the innate immune system in these compartments to initiate antibody‐dependent cellular cytotoxicity, complement‐dependent cytotoxicity, or antibody‐dependent cellular phagocytosis. For this reason, ITs are predicted to be much more effective at depletion in target tissues than therapeutic antibodies. In fact, it was reported that CD3 IT was more effective than the mAb to deplete skin T cells (Watanabe *et al.*, 2015). For these reasons, we speculate that our CCR4 IT and the bispecific IT will be more effective than FDA‐approved mogamulizumab for treatment of refractory or relapsed CTCL. Combined treatment with mogamulizumab and the CCR4 IT or the bispecific IT might be another approach for targeting relapsed and refractory CTCL. In addition, the bispecific IT will target more broad CTCL patients including CD25^+^ or/and CCR4^+^ cases. Therefore, the bispecific IT might be a more promising agent for treatment of refractory and relapsed CTCL patients.

As shown in Fig. 5 and Tables 2 and 3, CCR4‐IL2 bispecific IT prolonged the tumor‐bearing animal survival significantly longer than IL2‐CCR4 bispecific IT (median survival days of 69 vs 57). The only difference between these two bispecific ITs is the order of IL2 and anti‐human CCR4 scFv in the C terminus of DT390 (either IL2‐CCR4 or CCR4‐IL2; see Fig. 1). Histological analysis demonstrated that two tumor cell areas (Fig. 7D) were identified in the examined section of IL2‐CCR4 bispecific IT‐treated animal liver. In contrast, no tumor cell area (Fig. 7E) was identified in the examined section of the CCR4‐IL2 bispecific IT‐treated animal liver. These histological data might explain why CCR4‐IL2 IT leads to longer animal survival than IL2‐CCR4 IT (Fig. 5). However, the detailed molecular mechanism remains unknown. Further studies are required to delineate the etiology of prolonged survival in one bispecific IT arrangement over the other.

Immunogenicity is a concern with IT therapy in the setting of multiple‐course treatments. CTCL patients have been reported to become immunodeficient as their disease progresses. Possible reasons include immunosuppressive cytokine secretion, dysregulation of immunoregulatory protein expression by the tumor cells, and loss of T‐cell receptor repertoire complexity. It is also possible that some CTCL cells may act as Tregs and effectively suppress the host antitumor immunity (Krejsgaard *et al.*, 2012). This might be the reason that FDA approved Ontak® for multiple‐course administration (9 or 18 µg·kg^−1^·day^−1^ by intravenous infusion over 30–60 min for five consecutive days every 21 days for eight cycles). In December 2018, DT‐based human IL‐3 fusion toxin ELZONRIS™ (tagraxofusp‐erzs; SL‐401; Stemline Therapeutics, Inc., New York, NY, USA) was approved by FDA at 12 µg·kg^−1^, intravenously over 15 min once daily on days 1–5 of a 21‐day cycle for multiple consecutive cycles for the treatment of blastic plasmacytoid dendritic cell neoplasm in adult and pediatric patients 2 years and older, in both treatment‐naïve and previously treated populations (Alkharabsheh and Frankel, 2019). We speculate that the CCR4 IT and the bispecific IT could also be administered for multiple‐course treatment of CTCL, as the same truncated diphtheria toxin DT390 was used.

## 5 Conclusions

We have demonstrated that the CCR4 IT is more effective than the IL2 fusion toxin and the IL2‐CCR4/CCR4‐IL2 bispecific IT is more effective than the CCR4 IT or the IL2 fusion toxin alone to human CD25^+^CCR4^+^ CTCL. We believe that the bispecific IT is a novel promising therapeutic drug candidate for treatment of refractory and relapsed CTCL patients.

## Conflict of interest

The authors declare no conflict of interest.

## Author contributions

HW and ZhaW primarily performed the experiments and data analysis and participated in writing the manuscript; HZ and ZQ participated in the experiments; ACJ participated in writing the manuscript; ER analyzed the pathology data; CAH, DM, and EAP participated in the data analysis and writing the manuscript; and ZhiW primarily designed the project, analyzed the data, and wrote the manuscript.

## Supporting information


**Fig. S1.** Flow cytometry binding affinity analysis and *K*_d_ determination of the bispecific immunotoxins to human CD25 and CCR4 double negative Jurkat cell line, human CD25 single positive SR cell line and human CCR4 single positive CCL‐119 cell line.

**Fig. S2.** *In vitro* efficacy analysis of the bispecific immunotoxins to human CD25 and CCR4 double negative Jurkat cell line, human CD25 single positive SR cell line and human CCR4 single positive CCL‐119 cell line.

**Fig. S3.** Liver necropsy examination (repetition) of the representative tumor‐bearing *NSG* mice.
