# Supplementary material for: Bispecific human IL2‐CCR4 immunotoxin targets human cutaneous T‐cell lymphoma
Source: Mol Oncol. 2020 Mar 13;14(5):991–1000. doi: 10.1002/1878-0261.12653 (PMC7191189; doi:10.1002/1878-0261.12653)
Supplement: Supplementary file 1 — Fig. S1. Flow cytometry binding affinity analysis and K d determination of the bispecific immunotoxins to human CD25 and CCR4 double negative Jurkat cell line, human CD25 single positive SR cell line and human CCR4 single positive CCL‐119 cell line. [file MOL2-14-991-s001.pdf]

Figure S1A

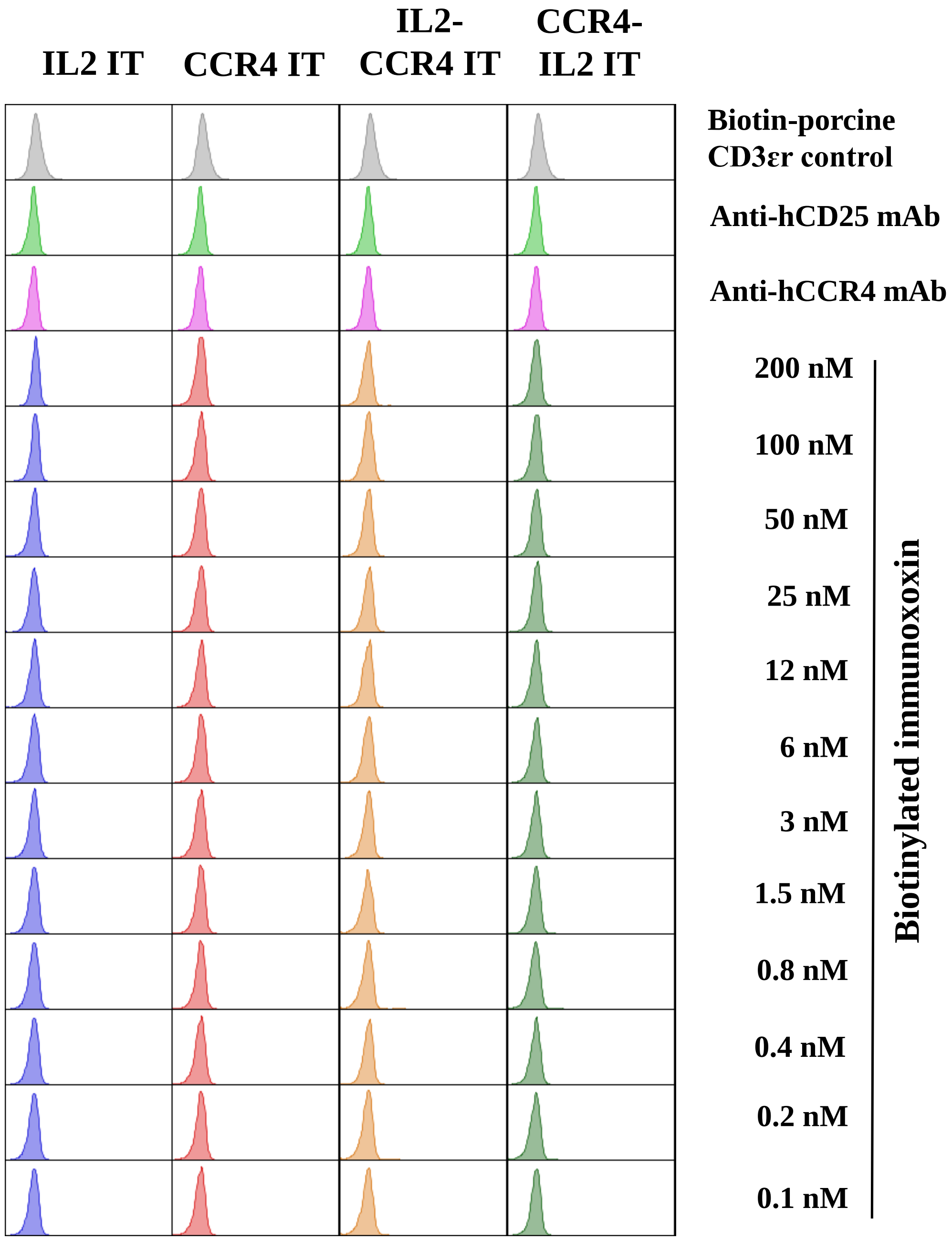
IL2 IT
CCR4 IT
IL2-CCR4 IT
CCR4-IL2 IT
Biotin-porcine CD3εr control
Anti-hCD25 mAb
Anti-hCCR4 mAb
200 nM
100 nM
50 nM
25 nM
12 nM
6 nM
3 nM
1.5 nM
0.8 nM
0.4 nM
0.2 nM
0.1 nM
Biotinylated immunoxoxin

Figure S1B

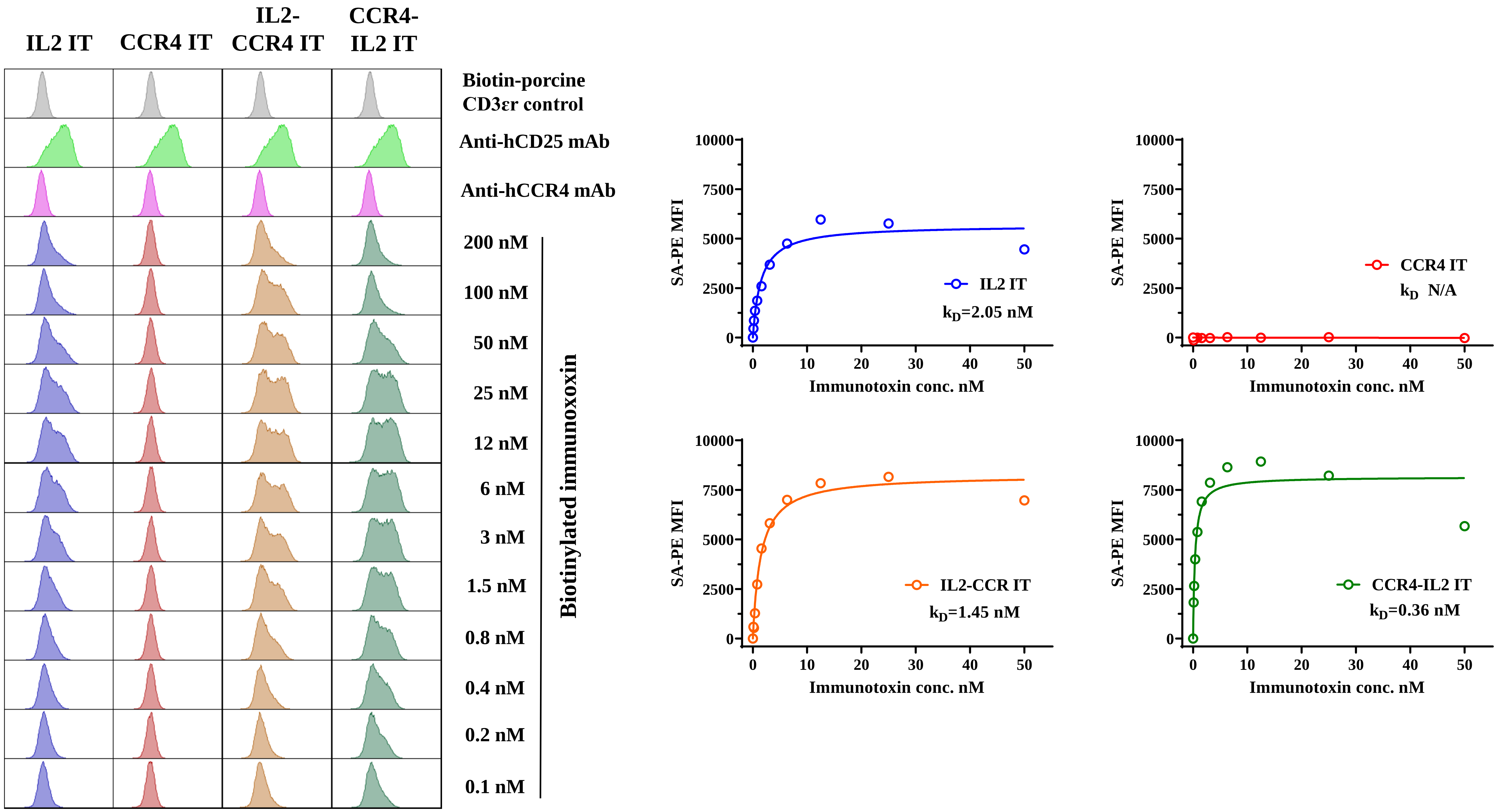
IL2 IT
CCR4 IT
IL2-CCR4 IT
CCR4-IL2 IT
Biotin-porcine CD3εr control
Anti-hCD25 mAb
Anti-hCCR4 mAb
200 nM
100 nM
50 nM
25 nM
12 nM
6 nM
3 nM
1.5 nM
0.8 nM
0.4 nM
0.2 nM
0.1 nM
Biotinylated immunoxoxin
SA-PE MFI
Immunotoxin conc. nM
IL2 IT
k_D=2.05 nM
CCR4 IT
k_D N/A
IL2-CCR IT
k_D=1.45 nM
CCR4-IL2 IT
k_D=0.36 nM

Figure S1C

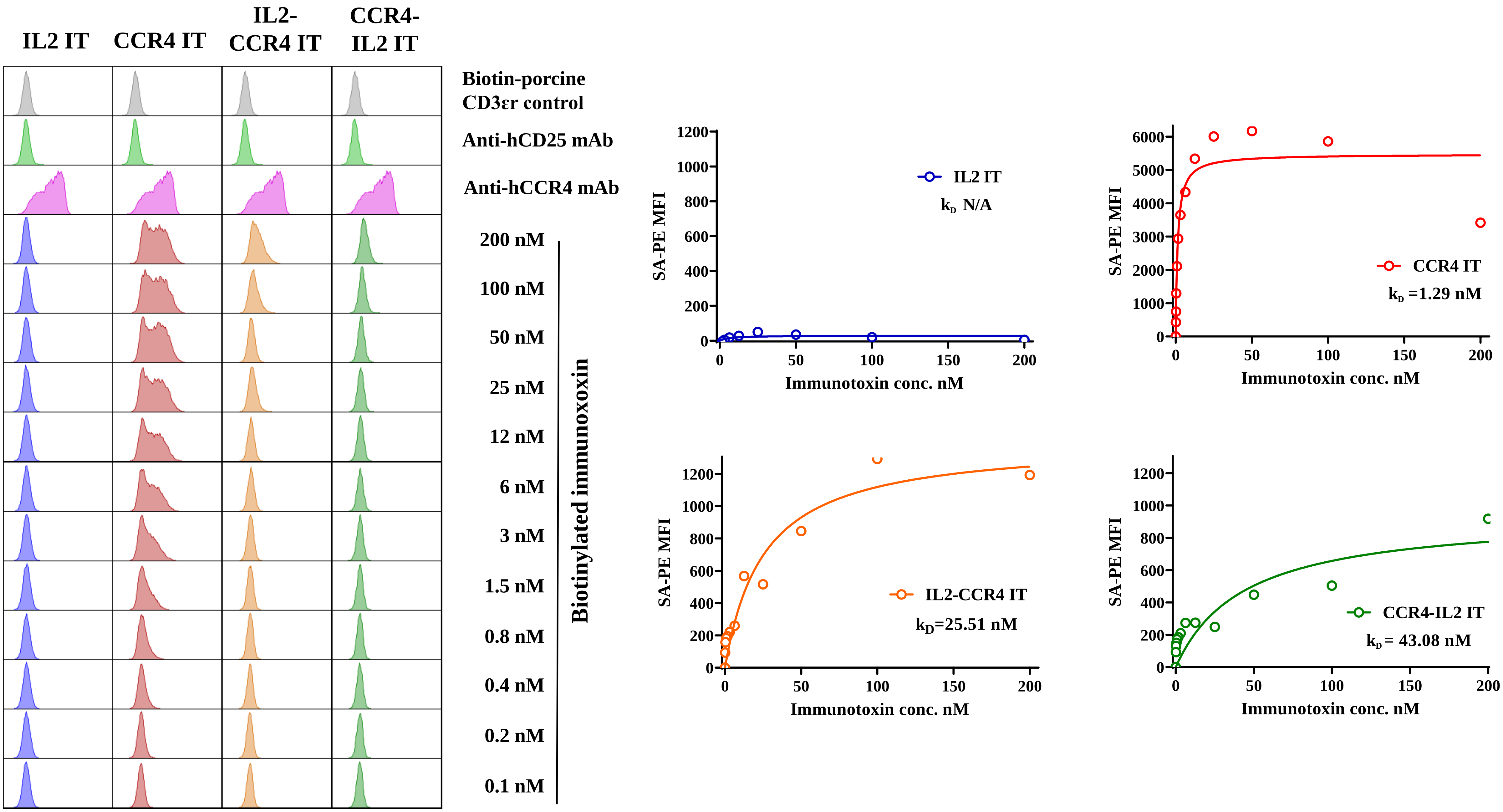

IL2 IT
CCR4 IT
IL2-CCR4 IT
CCR4-IL2 IT
Biotin-porcine CD3εr control
Anti-hCD25 mAb
Anti-hCCR4 mAb
200 nM
100 nM
50 nM
25 nM
12 nM
6 nM
3 nM
1.5 nM
0.8 nM
0.4 nM
0.2 nM
0.1 nM
Biotinylated immunoxoxin
SA-PE MFI
Immunotoxin conc. nM
IL2 IT
kD N/A
CCR4 IT
kD =1.29 nM
IL2-CCR4 IT
kD=25.51 nM
CCR4-IL2 IT
kD = 43.08 nM

Figure S1D

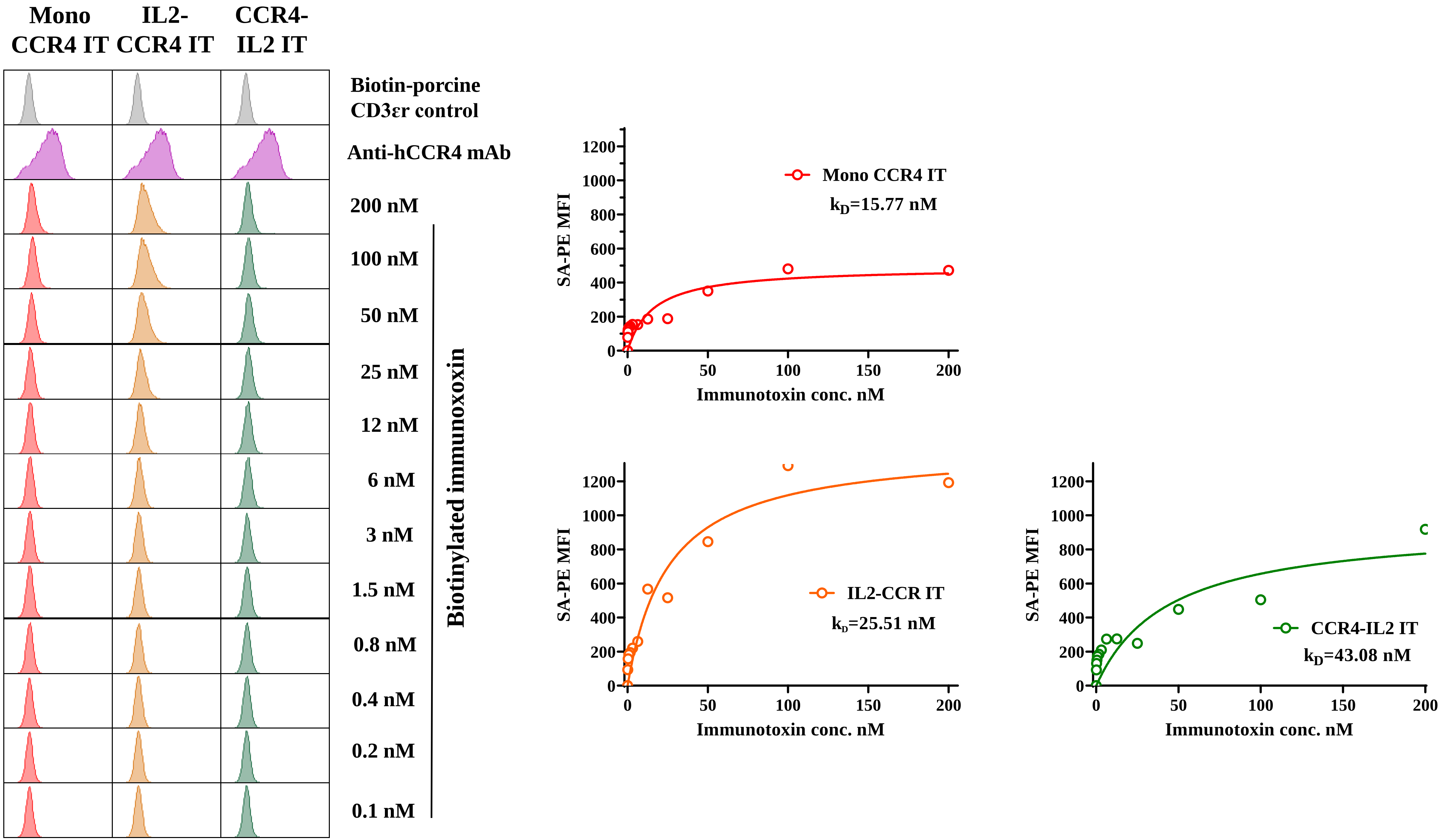

Mono CCR4 IT
IL2-CCR4 IT
CCR4-IL2 IT
Biotin-porcine CD3εr control
Anti-hCCR4 mAb
200 nM
100 nM
50 nM
25 nM
12 nM
6 nM
3 nM
1.5 nM
0.8 nM
0.4 nM
0.2 nM
0.1 nM
Biotinylated immunoxoxin
SA-PE MFI
Immunotoxin conc. nM
Mono CCR4 IT
kD=15.77 nM
IL2-CCR IT
kD=25.51 nM
CCR4-IL2 IT
kD=43.08 nM

Figure S1. Left panel: Flow cytometry binding affinity analysis of the biotinylated IL2-CCR4 or CCR4-IL2 bispecific immunotoxins to **A)** human CD25 and CCR4 double negative Jurkat cell line; **B)** human CD25 single positive SR cell line; **C-D)** human CCR4 single positive CCL-119 cell line. Biotinylated IL2 fusion toxin alone, foldback-diabody anti-human CCR4 immunotoxin alone (CCR4 IT) and monovalent anti-human CCR4 immunotoxin alone (mono CCR4 IT) were included as controls. Fluorescein-mouse anti-human/rat CCR4 mAb and FITC-mouse anti-human CD25 mAb were used as positive controls. Biotin-labeled porcine CD3-εγ (Peraino et al., 2012) was included as a negative control for background due to protein biotinylation. The data are representative of three individual experiments. **Right panel** (in Figure S1B-D)**:** K_D determination using flow cytometry and nonlinear regression, saturation binding equation by GraphPad Prism. MFI was plotted over a wide range of concentrations of the biotinylated **1)** IL2 fusion toxin alone; **2)** foldback diabody anti-human CCR4 immunotoxin alone (CCR4 IT); **3)** IL2-CCR4 bispecific immunotoxin; **4)** CCR4-IL2 bispecific immunotoxin; **5)** monovalent anti-human CCR4 immunotoxin alone (Mono CCR4 IT, only in Figure S1D). The nonlinear regression fit shown was based the equation $Y = Bmax * X/(K_D + X)$, where Y = MFI at the given biotinylated immunotoxin concentration after subtracting off the background; X = biotinylated immunotoxin concentration; Bmax = the maximum specific binding in the same units as Y.
