# Supplementary material for: Bispecific human IL2‐CCR4 immunotoxin targets human cutaneous T‐cell lymphoma
Source: Mol Oncol. 2020 Mar 13;14(5):991–1000. doi: 10.1002/1878-0261.12653 (PMC7191189; doi:10.1002/1878-0261.12653)
Supplement: Supplementary file 2 — Fig. S2. In vitro efficacy analysis of the bispecific immunotoxins to human CD25 and CCR4 double negative Jurkat cell line, human CD25 single positive SR cell line and human CCR4 single positive CCL‐119 cell line. [file MOL2-14-991-s002.pdf]

Figure S2A

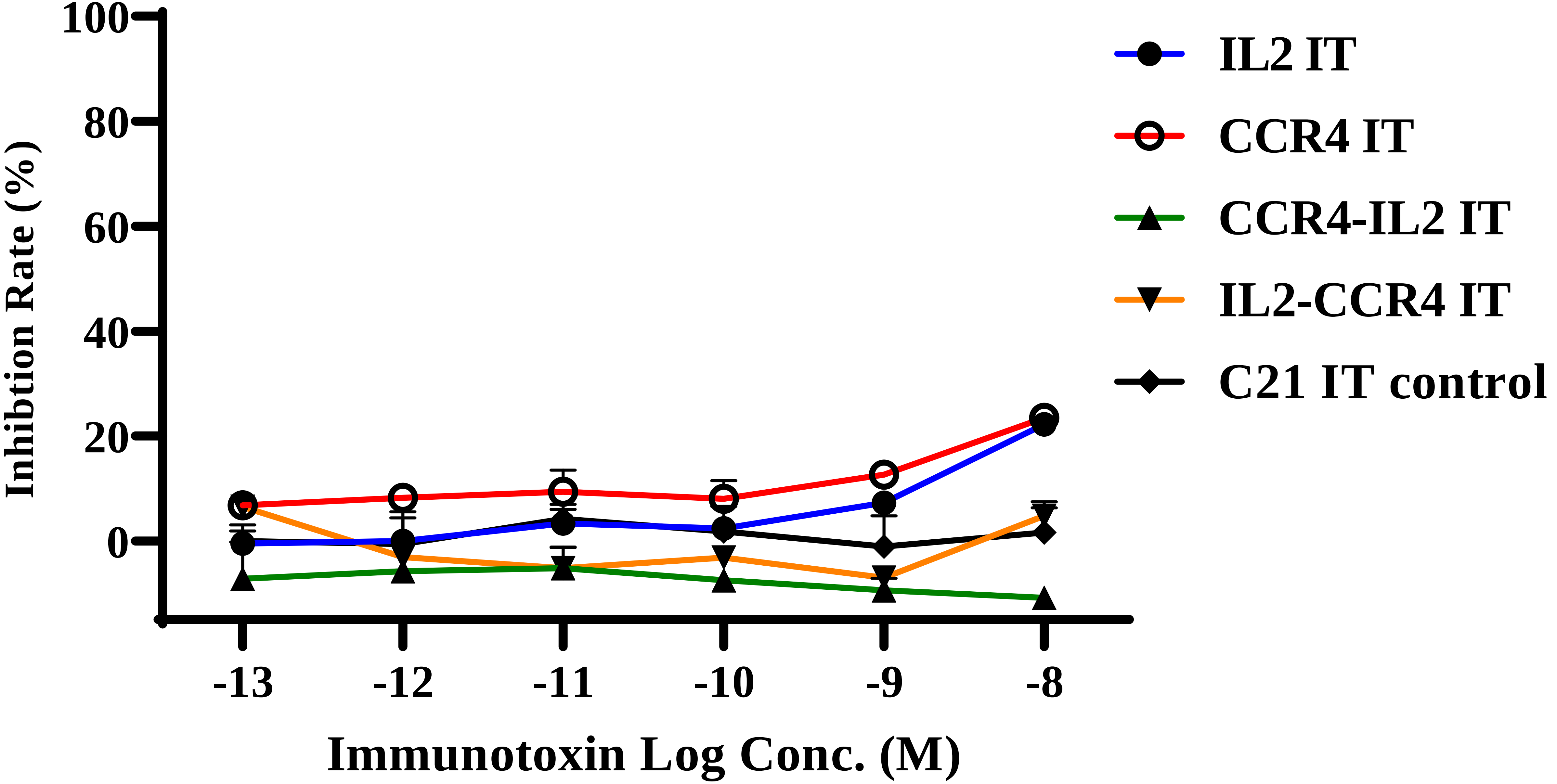

100
80
60
40
20
0
Inhibtion Rate (%)
-13
-12
-11
-10
-9
-8
Immunotoxin Log Conc. (M)
IL2 IT
CCR4 IT
CCR4-IL2 IT
IL2-CCR4 IT
C21 IT control

Figure S2B

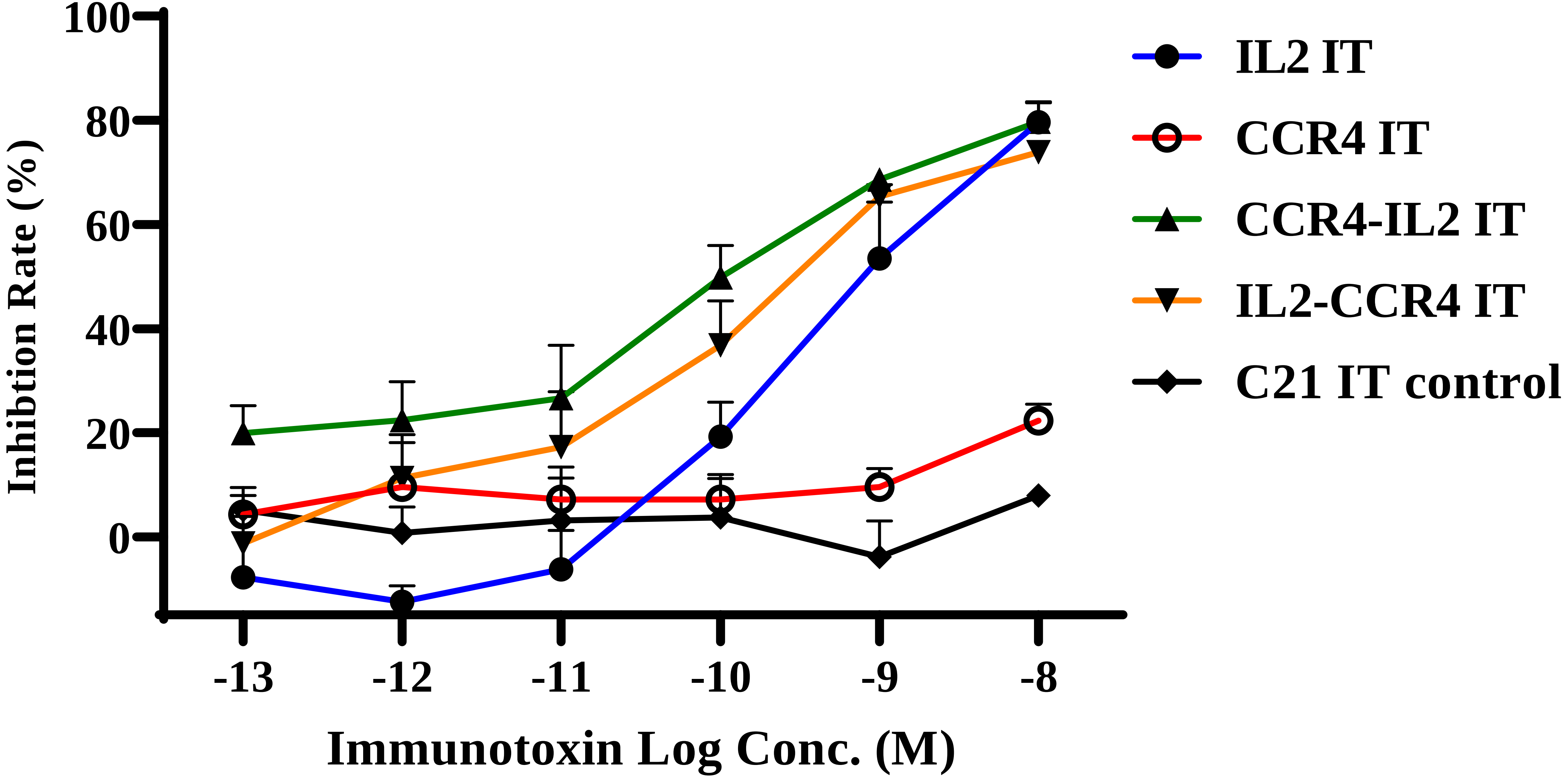

Inhibtion Rate (%)
100
80
60
40
20
0
-13
-12
-11
-10
-9
-8
Immunotoxin Log Conc. (M)
IL2 IT
CCR4 IT
CCR4-IL2 IT
IL2-CCR4 IT
C21 IT control

Figure S2C

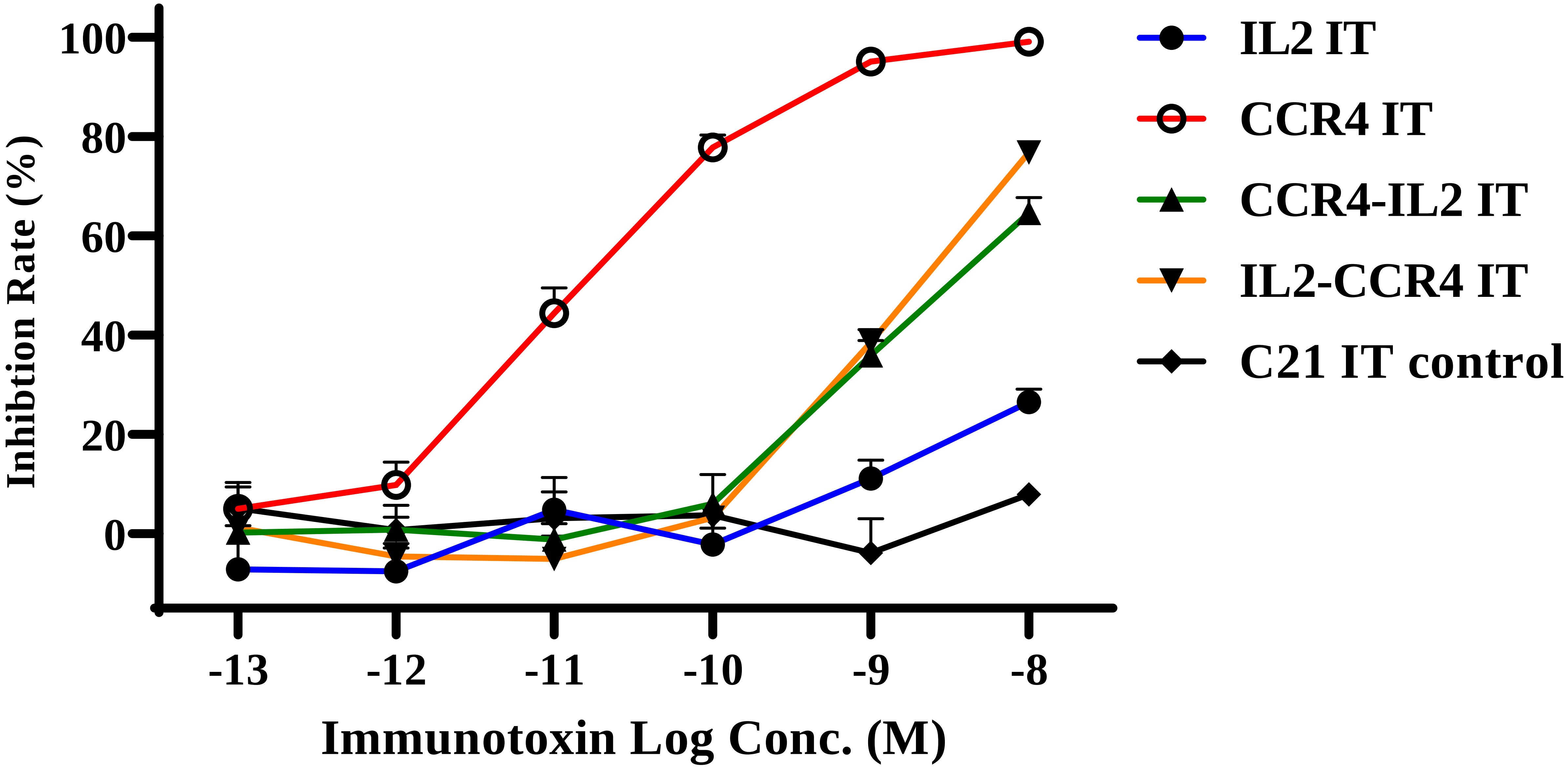
Inhibtion Rate (%)
100
80
60
40
20
0
-13
-12
-11
-10
-9
-8
Immunotoxin Log Conc. (M)
IL2 IT
CCR4 IT
CCR4-IL2 IT
IL2-CCR4 IT
C21 IT control

Figure S2D

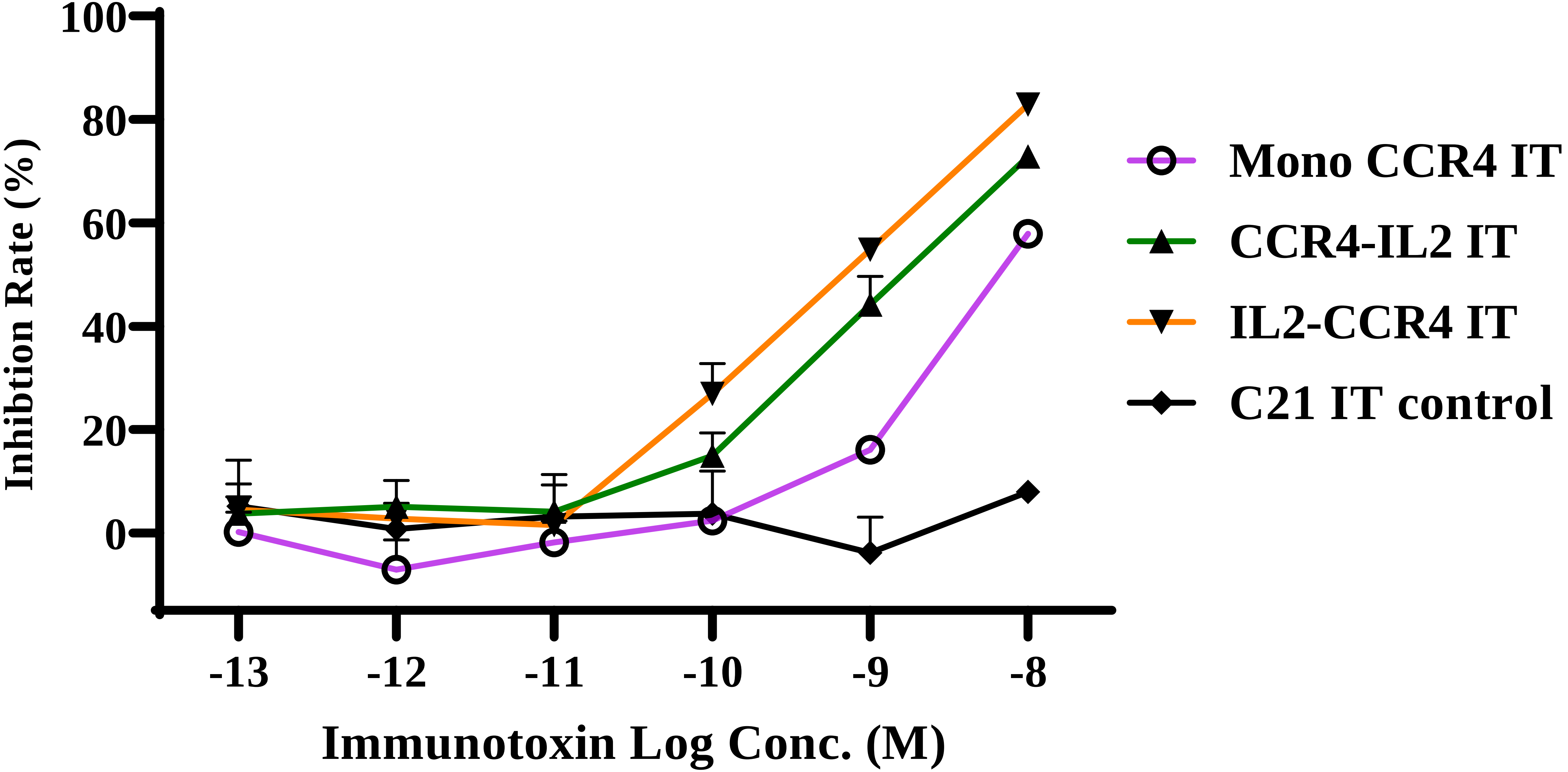
Inhibtion Rate (%)
100
80
60
40
20
0
-13
-12
-11
-10
-9
-8
Immunotoxin Log Conc. (M)
Mono CCR4 IT
CCR4-IL2 IT
IL2-CCR4 IT
C21 IT control

Figure S2. *In vitro* efficacy analysis of the bispecific immunotoxins using CellTiter-Glo® Luminescent Cell Viability Assay (Promega, cat# G7571) to **A)** human CD25 and CCR4 double negative Jurkat cell line; **B)** human CD25 single positive SR cell line; **C and D)** human CCR4 single positive CCL-119 cell line. C21 immunotoxin as negative control (Figure S2A-D, black line); IL2 fusion toxin alone (Figure S2A-C, blue line); foldback diabody anti-human CCR4 immunotoxin alone (CCR4 IT) (Figure S2A-C, red line); IL2-CCR4 bispecific immunotoxin (Figure S2A-D, orange line); CCR4-IL2 bispecific immunotoxin (Figure S2A-D, green line); monovalent anti-human CCR4 immunotoxin (mono CCR4 IT) (Figure S2D, purple line). Y-axis: inhibition rate of the cell viability by determining the number of viable cells based on the quantification of the ATP present. X-axis: plated immunotoxin concentration. Cycloheximide (1.25 mg/mL) was used as a positive control. The negative control contained cells without immunotoxin. Data were from three independent assays. Statistical analysis was performed using two-way ANOVA (n=3). Error bars indicate ±SD.
