# Supplementary material for: Bispecific human IL2‐CCR4 immunotoxin targets human cutaneous T‐cell lymphoma
Source: Mol Oncol. 2020 Mar 13;14(5):991–1000. doi: 10.1002/1878-0261.12653 (PMC7191189; doi:10.1002/1878-0261.12653)
Supplement: Supplementary file 3 — Fig. S3. Liver necropsy examination (repetition) of the representative tumor‐bearing NSG mice. [file MOL2-14-991-s003.pdf]

Figure S3

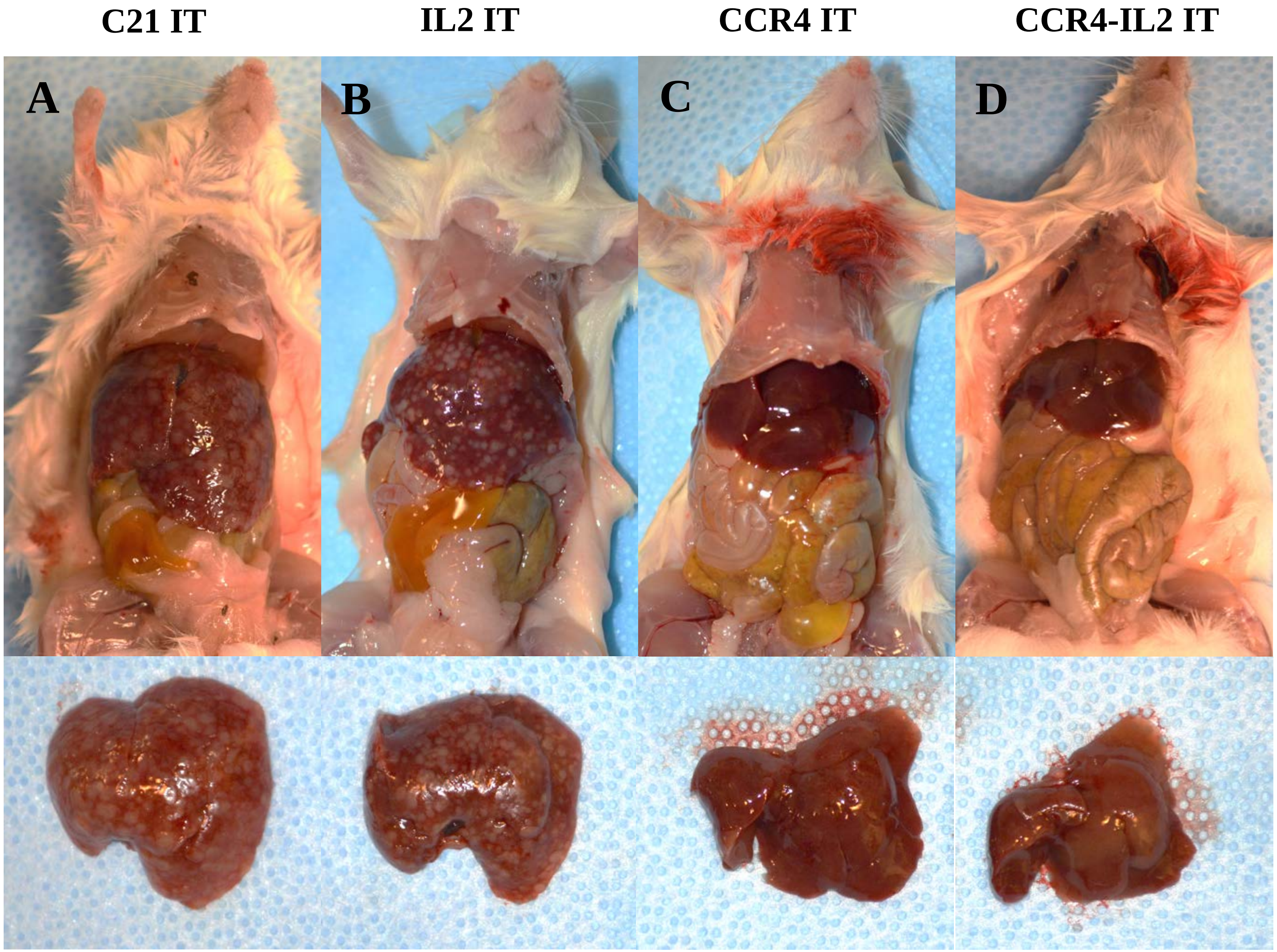

Figure S3. Liver necropsy examination (repetition) of the representative tumor-bearing *NSG* mice at day 21 from **A)** C21 immunotoxin group; **B)** IL2 fusion toxin alone group; **C)** CCR4 immunotoxin alone group; **D)** CCR4-IL2 bispecific immunotoxin group.
